# Effect of Radiation-Induced Cross-Linking on Thermal Aging Properties of Ethylene-Tetrafluoroethylene for Aircraft Cable Materials

**DOI:** 10.3390/ma14020257

**Published:** 2021-01-07

**Authors:** Xiaodong Zhang, Fei Chen, Zhimin Su, Taiping Xie

**Affiliations:** 1 School of Materials Science and Engineering, Yangtze Normal University, Chongqing 408100, China; 2008zhangdong@163.com; 2 Department of Chemical Engineering and Safety, BinZhou University, Binzhou 256603, Shandong, China; 3 Chongqing Academy of Chinese Materia Medica, Chongqing 400065, China; Ton_ly@sina.com

**Keywords:** ETFE, irradiation, aging, mechanical properties, crystallization kinetics

## Abstract

The effects of electron beam irradiation on ethylene-tetrafluoroethylene copolymer (ETFE) were studied. Samples were irradiated in air at room temperature by a universal electron beam accelerator for various doses. The effect of irradiation on samples and the cross-linked ETFE after aging were investigated with respect to thermal characteristics, crystallinity, mechanical properties, and volume resistivity using thermo-gravimetric analysis (TGA), differential scanning calorimeter (DSC), universal mechanical tester, and high resistance meter. TGA showed that thermal stability of irradiated ETFE is considerably lower than that of unirradiated ETFE. DSC indicates that crystallinity is altered greatly by cross-link. The analysis of mechanical properties, fracture surface morphology, visco-elastic properties and volume resistivity certify radiation-induced cross-linking is vital to aging properties.

## 1. Introduction

Fluoropolymers, thermoplastic polymers, have been widely applied for many years due to outstanding mechanical properties, cold resistance, high heat, electrical insulation, and chemical resistance. These materials confer a superior temperature resistance and have been used a lot for cable insulation across the aerospace field [1,2]. Although fluoropolymers have been extensively employed as insulation materials in spaceships and aircrafts, fluoropolymer, which is used for aerospace applications, is frequently decomposed on account of its rather low radiation resistance performance derived from fluoride precipitation after cosmic ray radiation [3,4]. Hence, there has been a substantial drop-off in mechanical properties, chemical resistance, electrical properties, thermal stability, surface properties, and other characteristics of perfluoropolymers where the body is exposed to rays [5,6] and the extent of degradation depends upon the radiation dose, dose rate, and energy of the incident radiation [7,8,9,10,11,12,13].

Ethylene-tetrafluoroethylene copolymer (ETFE), which occupies a special status among fluoropolymers, is a semi-crystalline polymer and is essentially a 1:1 alternating copolymer of ethylene and tetrafluoroethylene. ETFE has a higher radiation stability and exhibits superior mechanical properties, flexural modulus and creep resistance than its perfluorinated counterparts, i.e., poly (tetrafluoroethylene) (PTFE), poly (tetra fluoroethylene-co-hexafluoropropylene) (FEP), and poly (tetrafluoroethylene-co-pefluorovinylether) (PFA) [3,14,15,16,17] Oshima et al. [18] reported that polytetrafluoroethylene (PTFE) is extremely sensitive to radiation and undergoes a chain scission even at a very small radiation dose. Galante et al. [19] investigated the radiation tolerance of perfluoroalkoxy (PFA) under vacuum and in oxygen and found it to be higher than that of PTFE (the ratio being 10:1). This has made ETFE a particularly interesting candidate for the aerospace industry to replace other fluoropolymer and electron beam irradiation further enhances the radiation resistance, modulus, and mechanical properties of ETFE.

In the present paper, ETFE was modified by adding TAIC as cross-linking agent, and a network structure was formed upon electron beam irradiation. With a rapid development in the use of ETFE in the aircraft industry worldwide, a systematic investigation about the effects of irradiation with various doses on thermal stability and a series of properties after thermal aging was conducted imperatively. To the best of our knowledge, such detailed surveys have not been reported in any previous work. The objective of this study is to provide an in-depth understanding of cross-linking in ETFE for its industrial applications.

## 2. Experimental Procedures

### 2.1. Materials

ETFE (C-88AXMP) with MFI of 36g /10 min (290 °C/2.16 kg) and a specific gravity of 1.73, supplied by ASAHI GLASS Co., Ltd. (Tokyo, Japan), were used as the base polymer. The reagents used during cross-linked ETFE preparation were 1,3,5-tri-2-propenyl-1,3,5-triazine-2,4,6(1H,3H,5H)-trione(TAIC) and diantimony trioxide purchased from ALADDIN Co., Ltd. (Shanghai, China).

### 2.2. Sample Preparation

The curing agent (TAIC) and Sb_2_O_3_ as flame retardant were first manually mixed with ETFE. The ratio of ETFE, Sb_2_O_3_ and TAIC is 100:10:1. The blends were prepared using RM-200C parallel twin screw extruder (HAPU, Harbin, China) with a screw diameter of 20mm and an L/D ratio of 30:1. The torque of the twin screw extruder is from 0 to 250 N·m and the production capacity is 6 kg/h. The temperature of zones of feeding, compression, and metering was set at 230, 260 and 280 °C, respectively. The rotor speed was fixed at 40 rpm. The extrudates in the form of thin ribbon were immediately quenched in a water bath and repelletized in a subsequent operation. These were dried before the subsequent processing and characterization. In the following, the blends were compression molded in the form of a rectangular sheet with 1 mm of thickness by using flat-panel curing press (GT-7014-H30C of GOTECH, Dongguan, China) (temperature 285 °C and pressure 15 tons).

### 2.3. Irradiation

The samples were located on a pallet on a conveyer and irradiated in air at room temperature on a stepwise basis by using Kinwa High Technology Co., Ltd. of Changchun, China an acceleration voltage of 1.2 MeV and a dose rate of 2.4 × 10^4^ kGy/h. The samples were exposed to continuous multiple irradiation from 60 to 180 kGy by increasing the number of passes. Afterwards, the irradiated specimens were ensconced at room temperature for 48 h in order to minimize the effects of free radical in samples.

### 2.4. Ageing Experiments

Thermal aging was conducted in a box furnace (LY-2150, LIYI Co., Shenzhen, China) for times varying between 0 to 504 h in an oxygen environment. Because of thermal degradation play a dominant role in aging reaction with temperature increasing. Therefore, aging experiments was achieved at temperatures of 230 °C, which is the limiting temperature for maintaining the crystal structure.

### 2.5. Measurements

**Figure 1:**
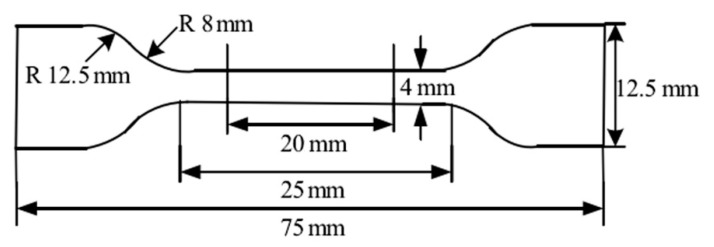
The detailed dimensions of tensile tests of the samples.

The thermal stabilities of specimens which were irradiated respectively at 60, 120, 180 kGy and pure ETFE were measured under nitrogen atmosphere with a Netzsch TGA 209C thermogravimetric analyzer (Selb, Germany) at a heating rate of 10 °C/min^−1^. Thermograms of heating of ETFE samples were characterized at a certain heating rate using a DSC 204 instrument (Netzsch, Wittelsbacherstr, Germany) from 50 to 300 °C, maintained isothermally at 300 °C for 3 min, cooled from 300 to 40 °C. The heat of melting was calculated from the areas under the melting peaks. The degree of crystallinity was calculated using the following Equation (1):

(1) $$Xc=\frac{∆H_{m}}{∆H_{100\%}}\times 100\%$$

where $∆H_{m}$ is the heat of melting of ETFE films which is proportional to the area under the melting peak and $∆H_{100\%}$ is the heat of melting of 100% crystalline ETFE polymer ($∆H_{100\%}$ = 288 J/g). The whiteness of specimens which were irradiated respectively at 60, 120, 180 kGy were carried out with whiteness meter (DRK130A, Drick, Jinan, China) according to China State Standard GB/T 15595-2008. The tensile tests of the samples were recorded with Universal Testing Machine (AI-3000 of GOTECH, DongGuan, China) at speed of 50 mm/min, the shape and size of the samples according to type I in the China State Standard GB/T1040.2-2006. The detailed dimensions are shown in the Figure 1. At least five specimens of each composition were tested, and the average values were recorded. Volume resistivity of samples with 0.5~1 mm of thickness was measured by using ZC36 type high resistance meter (BELL Analytical Instruments Co., Ltd., DaLian, China) according to GB1410-78. The fracture surface morphology of the samples which fractured in tensile strength experiments was observed using scanning electron microscope (SEM, Hitachi S-4700, Tokyo, Japan). All sample surfaces were coated with a thin gold layer by plasma sputtering to avoid any charging effect due to non-conductivity of the polymer. The visco-elastic properties were measured with dynamic mechanical analysis (NETZSCH DMA 200, Selb, Germany) and a three-point bending configuration at a heating rate of 3°/min.

## 3. Results and Discussion

### 3.1. Investigations into Thermal Stability

**Figure 2:**
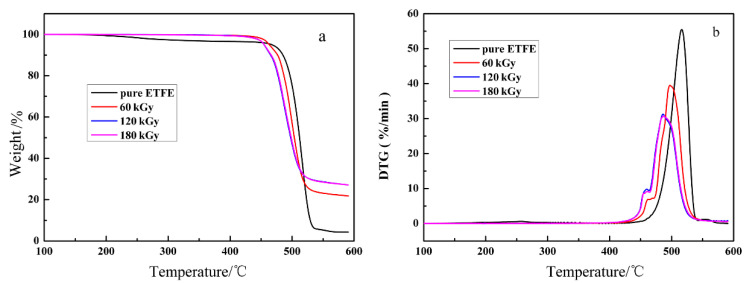
Thermo-gravimetric analysis (TGA) (**a**) and derivative thermogravimetry (DTG) (**b**) curves for samples pristine and irradiated at various doses.

With respect to applications, the thermal stability of the ETFE with TAIC and Sb_2_O_3_ is crucial. Therefore, the thermal stability of the specimens with different doses and unirradiated specimen was investigated by TGA and the results obtained are depicted in Figure 2a. The derivative thermogravimetry (DTG) curves are provided in Figure 2b. The results demonstrate that precursor (non-irradiated) pure ETFE exhibit a single-step degradation pattern with the transition at about 525 °C stemmed from the decomposition of molecular chains of pure ETFE. However, the degradation profile of irradiated ETFE with TAIC and Sb_2_O_3_ exhibit two distinct steps. The first stage peaks at a temperature of approximately 470 °C. The weight loss in the first stage remain unchanged with increasing dose are plotted in Figure 2b. Therefore, this behavior can explain the decomposition of TAIC molecules. The small amount of decomposition for pure ETFE in the temperature range of 250 to 450 °C is due to low molecular weight degradation. The most significant weight loss is attributed to decomposition of ETFE matrix as expected. The weight loss in the stage rises with increasing dose rested with the extent of structure changes rooted in these reactions as ETFE are exposed to irradiation process. During the irradiation process, scission reactions of C–F, C–H and C–C bonds occur in initial moment, resulting in the formation of macroradicals, which undergo the following competitive reactions [20]: (1) peroxidation by reaction with atmospheric oxygen generating in hydroperoxides after hydrogen abstraction from the neighboring ethylene molecules; (2) dehydrofluorination after C–C scission to form unsaturated structure and (3) dehydrofluorination and the subsequent formation of cross-linked structure by reaction with the adjacent macromolecular radical. Therefore, -CF_3_ side groups and branched structures generated during irradiation, which have lower thermal stability than the pure ETFE, will be increasing in the wake of the absorbed dose. This is significant potential for the decline in the thermal stability. On the other hand, the irradiated ETFE reveal higher carbon residue rate compared with the unirradiated ETFE. This can be explicated that network structures in irradiated ETFE are not decomposed completely at the temperature of 550 °C.

**Figure 3:**
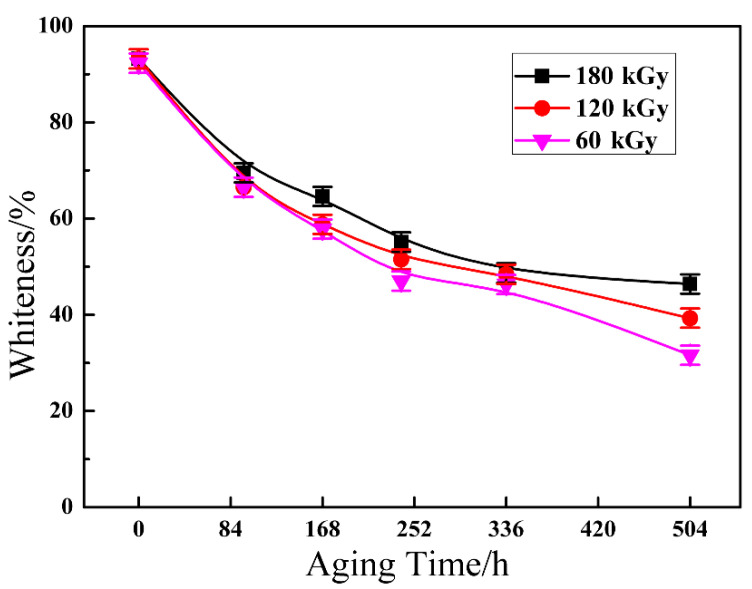
Whiteness as a function of aging time.

Despite the whiteness being a crucial index to evaluate the thermostability of ETFE in the application, studies relating to this issue are rare in the literature, especially regarding whiteness after aging. As shown in Figure 3, the whiteness of ETFE with various irradiation dose demonstrate a downtrend due to chain scission of the cross-linked system as an extension of aging time.

### 3.2. Kinetic Analysis of DSC

As we all know, ETFE is a semi-crystalline thermoplastic polymer with a high degree of crystallinity. As a consequence, the cables of ETFE insulation properties depend on these crystallization behaviors to a certain extent. On the other side, the processing approach of treating ETFE with irradiated matters transforms the crystallinity of such a semi-crystalline polymer in most cases. Therefore, it is indispensably important to survey the crystallization kinetics of ETFE due to the intimate connection between the properties and these crystallization behaviors.

**Figure 4:**
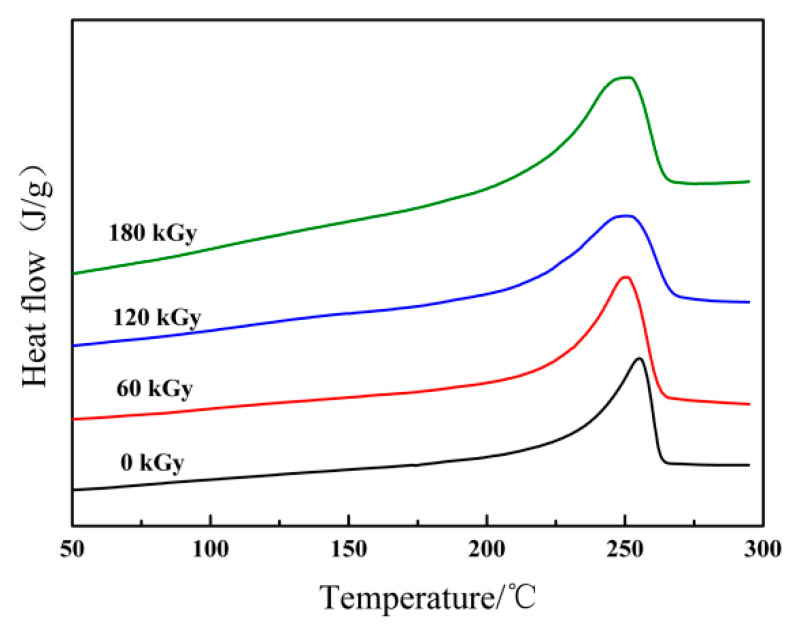
Typical melting thermograms of unirradiated ethylene-tetrafluoroethylene copolymer (ETFE) and irradiated ETFE at various doses.

With increasing irradiation dose, the increase is accompanied by transparency of ETFE specimens irradiated on oxygen-free conditions compared to unirradiated, opaque ETFE. The phenomenon manifests that the crystallinity of radiation-treated ETFE is reduced. In order to illustrate the structural-induced changes in ETFE, DSC measurements were conducted and the obtained thermograms were further analyzed to calculate the degree of crystallinity. Temperatures from the first heating run are plotted against absorbed dose in Figure 4 and the differential scanning calorimeter results shown in Table 1 for ETFE irradiated under oxygen-free atmosphere at room temperature. The melting temperature of irradiated polymers, which represents the crystallite sizes, displays a significant shift towards lower values with the irradiation dose increases. Moreover, the crystallinity degree of those irradiated ETFE have a parallel tendency of the melting temperatures. The result derived from non-isothermal DSC scans can contribute to cross-linking preventing the packing of chains and restricting the mobility of molecules. The crystallization in the amorphous region is impeded due to the augmenting of cross-linking effect. This eventually results in a remarkable decrease in the heat of melting and the degree of crystallinity.

**Figure 5:**
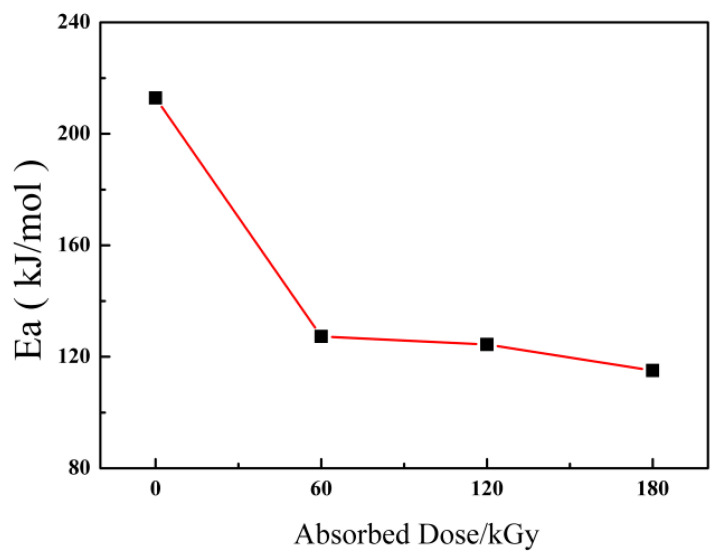
Activation energy of ETFE against absorbed irradiation doses.

On the other hand, non-isothermal DSC, which was performed at four heating rates: 5, 10, 15, 20 °C/min^−1^, was employed to study the non-isothermal oxidation induction temperature of the ETFE samples with different doses and unirradiated ETFE. The corresponding curves and results are depicted in Figures S1–S8 and Tables S1–S4 of supporting information. The Kissinger’s equation is used to estimate the activation energy (Δ*Ea*) of ETFE for clearer description of the variation of ETFE non-isothermal kinetics [21]:

(2) $$\frac{d[\ln(\beta /T_{\max}^{2})]}{d(1/T_{\max})}=-\frac{Ea}{R}$$

where *β* means the heating rate, *R* is the gas constant, and *T*_max_ is the crystallization peak temperature. The relationships of *Ea* versus absorbed dose are shown in Figure 5. As shown in Figure 5, the trend to decline in activation energy can be ascribed to the cross-linking effect obtained from irradiation process reduces the crystallization rate of ETFE.

**Figure 6:**
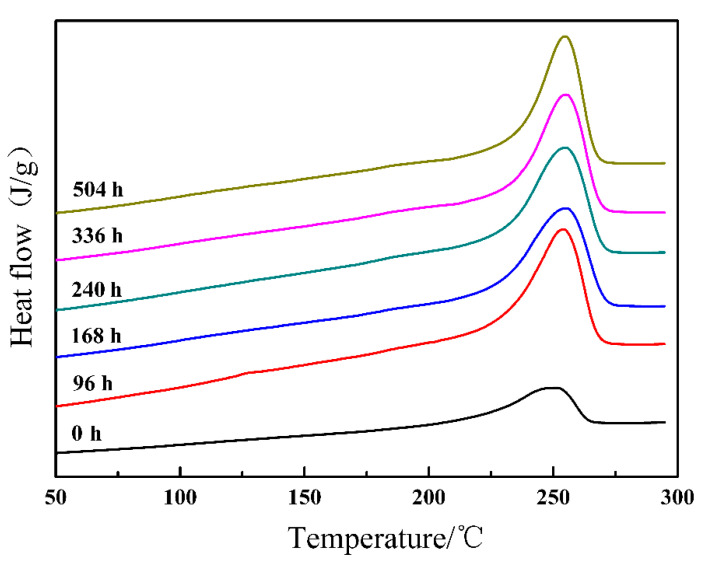
Typical melting thermograms for irradiated ETFE at 180 kGy against aging time.

Aging resistance of ETFE is a significant factor to consider as a potential candidate for aircraft cable. In order to evaluate effect of aging time on the crystallization behavior, aging experiments were conducted by adopting ETFE samples exposed to a dose of 180 kGy. The ETFE samples that undergo various aging time were measured with non-isothermal DSC. This can clearly be observed from the relationship between the aging time and the crystallinity degree shown in Figure 6 and the crucial parameters are summarized in Table 2. It is noteworthy that both the $∆H_{m}$ and the degree of crystallinity show a steady continually increasing trend with aging time. It can be affirmed that an increase in molecular mobility stemmed from the main chain degradation reaction and induced some of the broken polymer chains to recrystallize and others in the amorphous region to crystallize during the aging process.

### 3.3. Mechanical Properties

**Figure 7:**
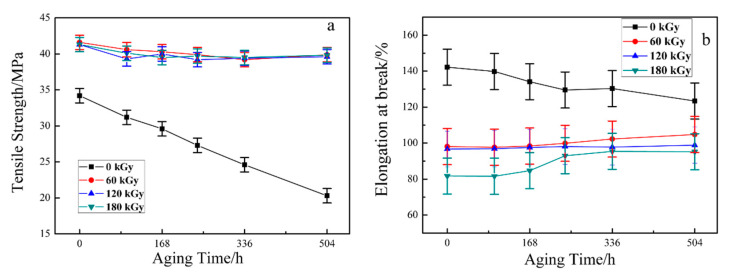
The effect of aging time on the mechanical properties of ETFE at various doses. (**a**) tensile strengthen; (**b**) elongation at break

The determination of mechanical properties after aging of induce-crosslinked ETFE is one of the most substantial subjects in estimating the performance of the cable materials based on ETFE. Mechanical properties of ETFE with various irradiation doses in tensile tests and elongation at break as a function of aging time are illustrated respectively in Figure 7a,b. As can be seen in Figure 7, the sharp reduction in the tensile strength and elongation at break of unirradiated ETFE with increasing aging time is expected given that chain scission and oxidation occur in the whole reaction. Furthermore, the obvious changes in the tensile strength and elongation at break of irradiated ETFE with different doses with increasing aging time are not observed. It can be asserted that the cross-linking structure formed in radiation processing effectively obstructs the main chain degradation during the aging process. Hence, the slight reduction of tensile strength and trifling augment in elongation at break are both due to the extent of cross-linking decline for chain scission during aging processing.

### 3.4. Fracture Morphology

**Figure 8:**
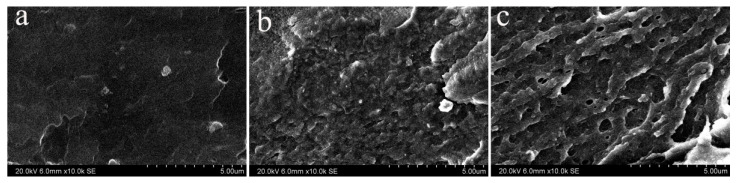
SEM micrographs of the fracture surface. (**a**) unirradiated ETFE after 504 h aging treatment; (**b**) irradiated ETFE after 504 h aging treatment at a dose of 60 kGy; (**c**) irradiated ETFE after 504 h aging treatment at a dose of 120 kGy.

In order to further investigate thoroughly structure-property relationship, themorphology of the fracture surfaces after tensile test was investigated by SEM. Figure 8 reveals the SEM fractographs of ETFE with variation irradiated dose after aging reaction. It is clear that the fracture surface of the unirradiated ETFE after 504 h aging treatment is very glossy, manifesting that the obstruction to crack propagation is very low after aging reaction and in accordance with its inferior mechanical strength. The micrographs of irradiated ETFE after 504 h aging treatment at a dose of 60 kGy and 120 kGy (Figure 8b,c) show deformed failure surfaces compared to unirradiated ETFE, which can be attributed to the formation of cross-linking network that act as crack-stoppers and can transform the direction of crack propagation when the specimen was loaded.

### 3.5. Volume Resistivity

**Figure 9:**
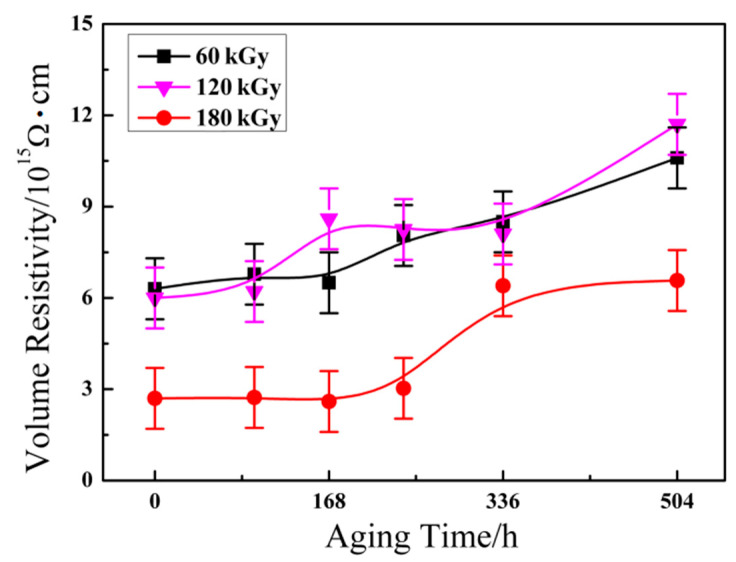
The effect of aging time on the volume resistivity of ETFE at various doses.

Volume resistivity of ETFE as the one of the most significant properties in the field of aviation insulation materials was investigated via high resistance meter. As is well known, the value of volume resistivity is influenced by the molecular structure in polymer; the higher crystallinity the higher volume resistivity. The orient macromolecules that induced high crystallinity in the matrix are bound firmly together, the compact structure can effectively reduce charge carriers (ions) mobility, and then give rise to an increase in volume resistivity. Figure 9 presents the variation of volume resistivity of ETFE with various irradiation dose against aging time. As mentioned above, the degree of crystallinity exhibits a steady continually increased trend during the aging process. Therefore, it is clearly seen that ETFE with various irradiation doses present a progressive enhancement in the volume resistivity when the aging time increases. The volume resistivity of irradiated ETFE with a dose of 180 kGy is far lower than specimens at the dose of 60 kGy and 120 kGy. The behavior can be explicated by the irradiated ETFE with a dose of 180 kGy possessing higher cross-linking degree, and the cross-linking effect hindering chain scission of cross-linked system during aging processing. Therefore, ETFE with a dose of 180 kGy exists in a more amorphous region and the structure causes the inferior volume resistivity.

### 3.6. Dynamic Mechanical Thermal Analysis

**Figure 10:**
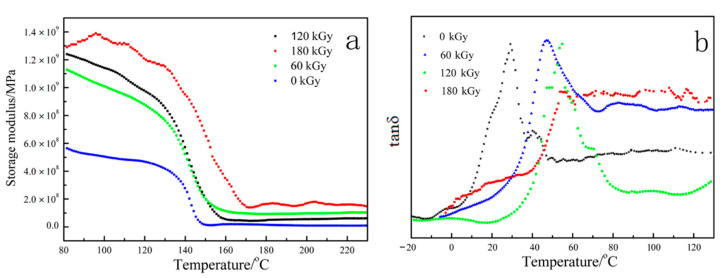
Dynamic mechanical behavior of ETFE treated with various irradiation doses after 336 h aging reaction.

DMA is an efficient method for providing significant information at the molecular level, helping us understand polymer mechanical behavior, in which the storage modulus (E’) and loss modulus (E”) of the sample under flexural load are measured against time, temperature or frequency of flexural load. As shown in Figure 10, the irradiated ETFE after aging reaction disposed over 336 h show a higher storage modulus and loss factor than nonirradiated sample after a period of 336 h aging treatment. Meanwhile, storage modulus and loss factor of irradiated ETFE mildly increase with an enhancive dose. It can be reasonably assumed that the cross-linking network of ETFE resulted from radiation reaction effectively enhancing the thermo-oxidative stability of the main chain during the aging processing. Therefore, irradiated ETFE with a varied dose still remains, to a certain extent, part of the cross-linking structure. It reduces the free volume, the segmental motion is hindered, and the macromolecular chains are in a frozen state, resulting in the superior storage modulus and Tg.

## 4. Conclusions

In this study, the irradiation effect on ETFE when TAIC was used as curing agent and the aging properties of irradiation cross-linked ETFE was systematically investigated. The TGA results demonstrate that the irradiation cross-linked ETFE is considerably lower than that of unirradiated ETFE. Meanwhile, a downtrend of thermostability of irradiation cross-linked ETFE was observed. Although the crystallinity of ETFE decreased with increasing dose, the improvement in the crystallinity of ETFE after aging was achieved in the nonisothermal DSC measure. Furthermore, mechanical properties and volume resistivity of cross-linked ETFE as a function of aging time was conveyed and the results indicate that cross-linking is a crucial factor for these properties.

## Figures and Tables

**Table 1:** Non-isothermal differential scanning calorimeter (DSC) scans results for the samples at different doses.

Absorbed Dose (kGy)	Onset (°C)	Peak (°C)	Tc (°C)	ΔH (J/g)	Xc (%)
0	232.8	255.1	237.9	41.1	36.3
60	225.2	250.5	237.0	34.4	30.3
120	214.4	250.7	238.0	31.0	27.4
180	221.6	251.1	238.2	24.1	21.3

**Table 2:** Non-isothermal DSC scan results for the samples irradiated at 180 kGy at various aging times.

Aging Time (h)	Onset (°C)	Peak (°C)	Tc (°C)	ΔH (J/g)	Xc (%)
0	221.6	251.1	238.2	24.1	21.3
96	229.1	254.0	241.5	27.9	24.6
168	226.3	255.2	241.0	29.9	26.4
240	229.6	255.0	241.7	30.4	26.8
336	232.5	255.1	241.5	32.4	28.6
504	234.7	254.9	241.7	31.6	27.9

## Data Availability

Data sharing is not applicable to this article.

## Supplementary Materials

The following are available online at https://www.mdpi.com/1996-1944/14/2/257/s1, Figure S1: DSC curves of the non-isothermal oxidation induction temperature of ETFE unirradiated at different heating rates, Figure S2: Linear relationship of ln$\left(\beta /T_{\max}^{2}\right)$ versus 1/$T_{\max}$, Figure S3: DSC curves of the non-isothermal oxidation induction temperature of ETFE absorbed 60 kGy at different heating rates, Figure S4: Linear relationship of ln$\left(\beta /T_{\max}^{2}\right)$ versus 1/$T_{\max}$, Figure S5: DSC curves of the non-isothermal oxidation induction temperature of ETFE absorbed 120 kGy at different heating rates, Figure S6. Linear relationship of ln$\left(\beta /T_{\max}^{2}\right)$ versus 1/$T_{\max}$ The values of Ea (124.37 kJ/mol) can be calculated ac-cording to the Kissinger’s equation, Figure S7: DSC curves of the non-isothermal oxidation induction temperature of ETFE absorbed 180 kGy at different heating rates, Figure S8: Linear relationship of ln$\left(\beta /T_{\max}^{2}\right)$ versus 1/$T_{\max}$, Table S1: Non-isothermal oxidation induction date obtained from the DSC scans at different heating rates, Table S2: Non-isothermal oxidation induction date obtained from the DSC scans at different heating rates, Table S3. Non-isothermal oxidation induction date obtained from the DSC scans at different heating rates, Table S4. Non-isothermal oxidation induction date obtained from the DSC scans at different heating rates.
