# Supplementary material for: Effect of Radiation-Induced Cross-Linking on Thermal Aging Properties of Ethylene-Tetrafluoroethylene for Aircraft Cable Materials
Source: Materials (Basel). 2021 Jan 7;14(2):257. doi: 10.3390/ma14020257 (PMC7825732; doi:10.3390/ma14020257)
Supplement: Supplementary file 1 [file materials-14-00257-s001.pdf]

Effect of Radiation Induced Crosslinking on Thermal Aging Properties of Ethylene-Tetrafluoroethylene for Aircraft Cable Materials

Xiaodong Zhang [1], Fei Chen [2,*] Zhimin Su [3] and Taiping Xie [1,*]

[1] School of Materials Science and Engineering, Yangtze Normal University, Chongqing 408100, China; 2008zhangdong@163.com
[2] Department of Chemical Engineering and Safety, BinZhou University, Binzhou 256603, Shandong, China.
[3] Chongqing Academy of Chinese Materia Medica, Chongqing 400065, China; Ton_ly@sina.com
* Correspondence: cfgxx@163.com (F.C.); deartaiping@163.com (T.X.)

The results of activation energy of ETFE experiments are shown in Figure S1.

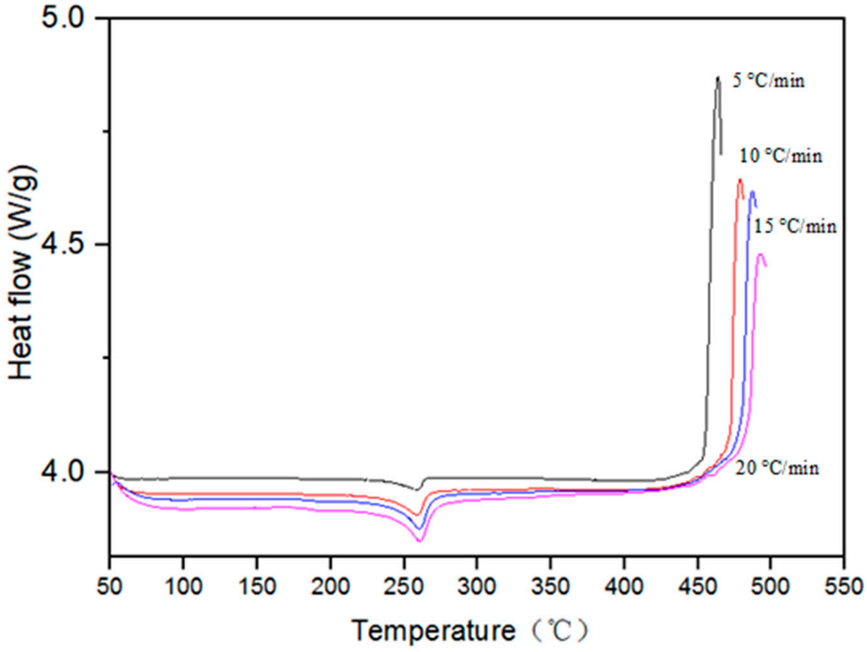

Figure S1. DSC curves of the non-isothermal oxidation induction temperature of ETFE unirradiated at different heating rates.

Table S1. Non-isothermal oxidation induction date obtained from the DSC scans at different heating rates.

Heating Rate/(°C/min)	Oxidation Induction Temperature /°C	Peak/°C
5	454.80	463.68
10	471.17	478.69
15	478.84	487.14
20	482.57	492.20

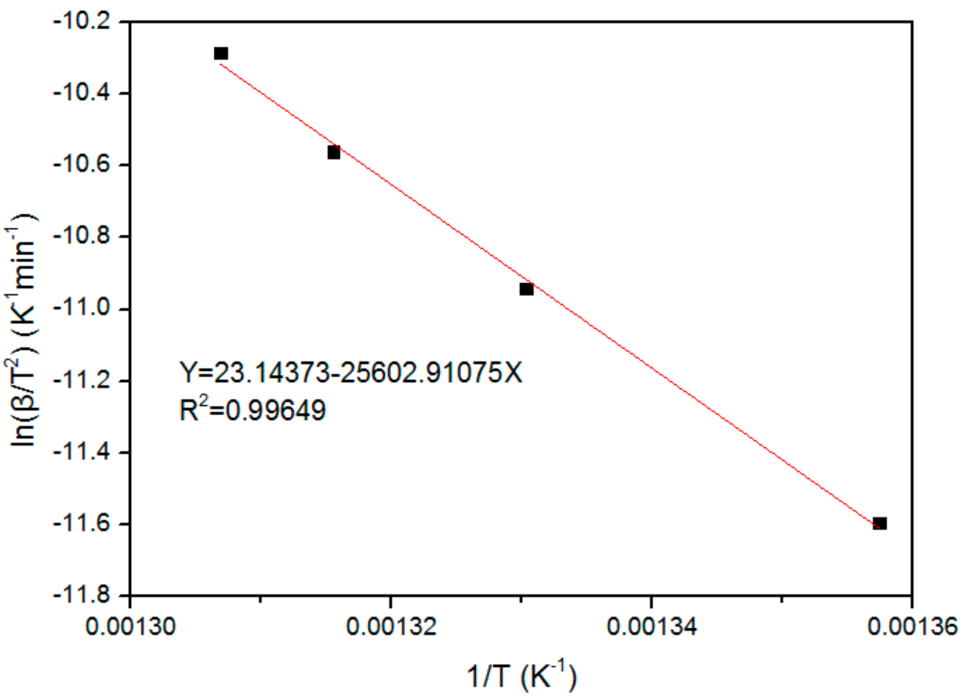

Figure S2. Linear relationship of $\ln(\beta / T_{max}^2)$ versus $\frac{1}{T_{max}}$.

The values of Ea (212.86 kJ/mol) can be calculated according to the Kissinger's equation.

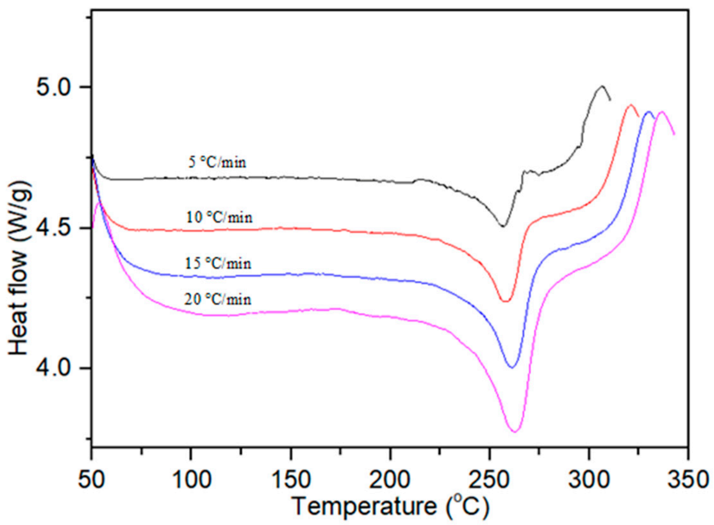

Figure S3. DSC curves of the non-isothermal oxidation induction temperature of ETFE absorbed 60 kGy at different heating rates.

Table S2. Non-isothermal oxidation induction date obtained from the DSC scans at different heating rates.

Heating Rate/(°C/min)	Oxidation Induction Temperature/°C	Peak/°C
5	294.98	306.82
10	307.15	322.16
15	314.57	330.11
20	320.17	337.51

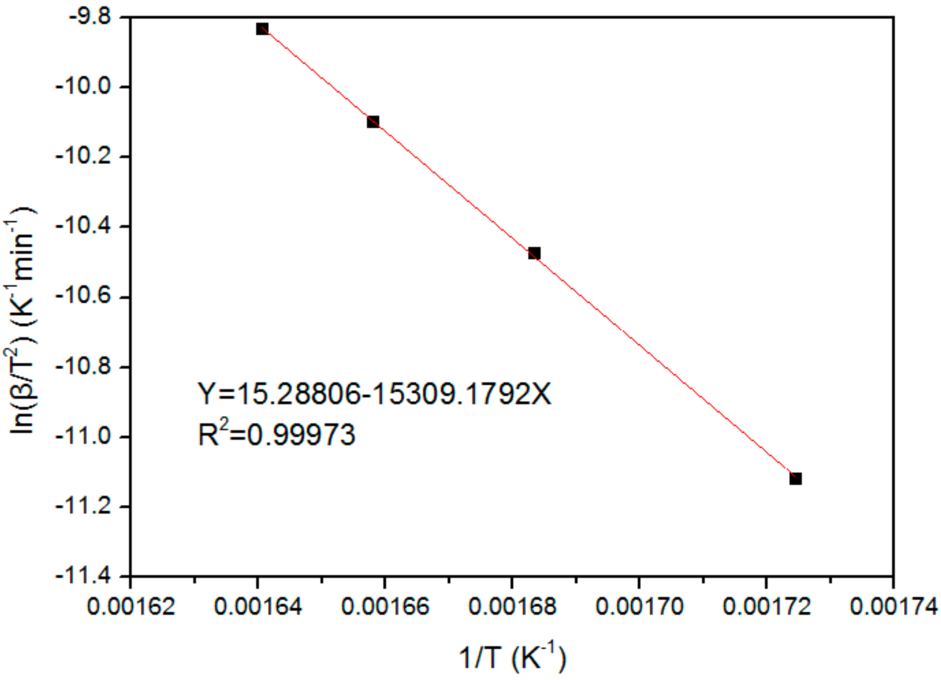

Figure S4. Linear relationship of $\ln(\beta / T_{max}^2)$ versus $\frac{1}{T_{max}}$.

The values of Ea (127.28 kJ/mol) can be calculated according to the Kissinger's equation.

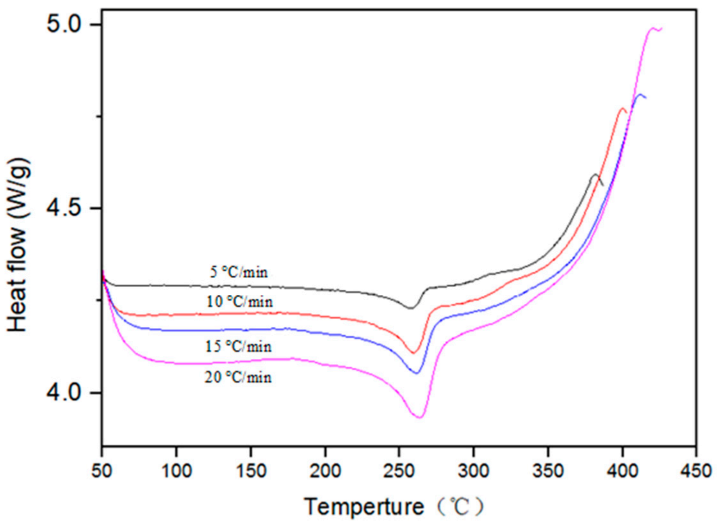

Figure S5. DSC curves of the non-isothermal oxidation induction temperature of ETFE absorbed 120 kGy at different heating rates.

Table S3. Non-isothermal oxidation induction date obtained from the DSC scans at different heating rates.

Heating Rate/(°C/min)	Oxidation Induction Temperature/°C	Peak/°C
5	348.92	381.80
10	364.82	399.55
15	375.10	411.48
20	388.85	420.46

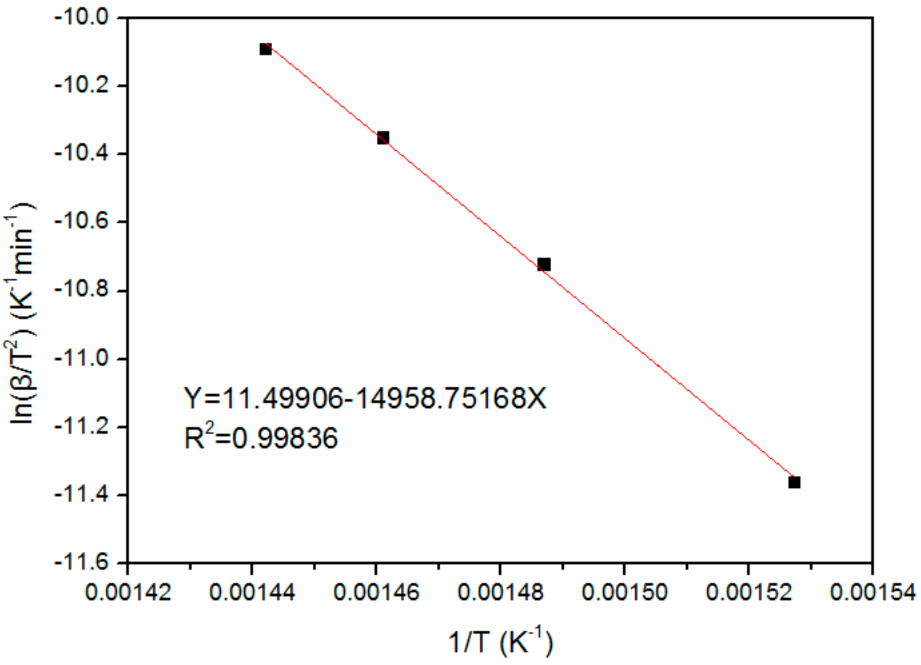

Figure S6. Linear relationship of $\ln(\beta / T_{max}^{2})$ versus $\frac{1}{T_{max}}$ The values of Ea (124.37 kJ/mol) can be calculated according to the Kissinger's equation.

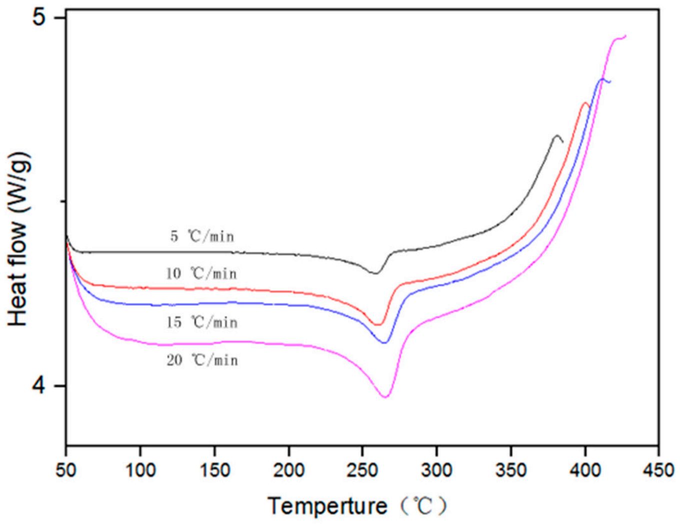

Figure S7. DSC curves of the non-isothermal oxidation induction temperature of ETFE absorbed 180 kGy at different heating rates.

Table S4. Non-isothermal oxidation induction date obtained from the DSC scans at different heating rates.

Heating Rate/(°C/min)	Oxidation Induction Temperature/°C	Peak/°C
5	346.42	380.98
10	365.94	400.00
15	376.70	411.42
20	380.81	423.15

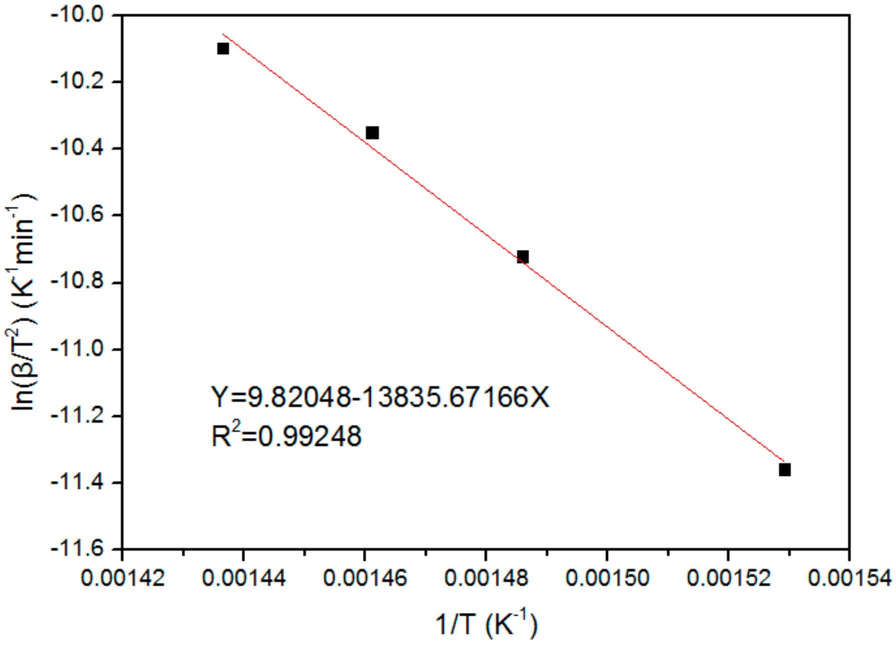

Figure S8. Linear relationship of $\ln(\beta / T_{max}^2)$ versus $\frac{1}{T_{max}}$.

The values of Ea (115.02 kJ/mol) can be calculated according to Kissinger's equation.
